# Case Report: Treatment of facial pyoderma gangrenosum with Upadacitinib

**DOI:** 10.3389/fmed.2025.1665013

**Published:** 2025-09-25

**Authors:** Gai Ge, Lirui Zhan, Meng Huang, Fei Su, Jinbo Chen, Jing Dong

**Affiliations:** ^1^Department of Dermatology, Traditional Chinese and Western Medicine Hospital of Wuhan, Tongji Medical College, Huazhong University of Science and Technology, Wuhan, China; ^2^Department of Dermatology, Wuhan No. 1 Hospital, Wuhan, China; ^3^Hubei Province & Key Laboratory of Skin Infection and Immunity, Wuhan, China

**Keywords:** facial ulcers, pyoderma gangrenosum, Janus kinase inhibitor, Upadacitinib, neutrophilic dermatosis

## Abstract

Pyoderma gangrenosum (PG) is a recurrent, painful, necrotizing ulcerative neutrophilic dermatosis. Facial PG (FPG) is a rare subtype of PG that is often misdiagnosed. A 20-year-old male with FPG showed improvement after treatment with a combination of prednisone and upadacitinib. Early diagnosis is crucial to avoid misdiagnosis.

## 1 Introduction

Pyoderma gangrenosum (PG) is a rare neutrophilic dermatosis that causes pustules and ulcerations (1). Its diagnosis and appropriate management are often delayed because of its rarity and the presence of numerous clinical variants. Although corticosteroids remain the primary first-line treatment for severe forms of PG, the use of Janus kinase inhibitors (JAKis) is promising (2, 3). To date, 31 cases of PG treated with JAKi have been reported and are presented in Table 1 (4–24). In this case report, the authors describe a 20-year-old man with long-standing, non-healing, and painful facial ulcers that were not responsive to multiple antibiotics and serial wound debridement. Treatment with Upadacitinib hydrate and a tapering course of oral prednisolone was initiated. However, FPG is a rare dermatological pathology lacking characteristic distinguishing features, making treatment challenging (25).

**Table 1:** Treatment of pyoderma gangrenosum with Janus kinase inhibitor.

No.	Authors	Age	Sex	Location	Comorbidity	Culprit condition	JAK inhibitory	Concomitant	Final outcome
1	Shanmugam et al. (4) 2013	63	M	Bilateral foot and ankle	Polycythemia vera, DVT, h/o colon cancer, Raynaud’s phe nomenon, Gottron’s papules	Wound debridement	Ruxolitinib	MTX (withdrawn)	Improved
2	Nasifoglu et al. (5) (2018)	64	F	Both legs	Polycythemia vera, myelofibrosis	ND	Ruxolitinib	ND	Healed
3	Kochar et al. (6) (2019)	49	F	Lower extremity	CD s/p colectomy	ND	Tofacitinib	ND	Healed
4	Kochar et al. (6) (2019)	24	M	Peristomal/around the stoma	Pouchectomy due to stricturing and fistulizing CD of the pouch	End ileostomy	Tofacitinib	Vedolizumab	Healed
5	Kochar et al. (6) (2019)	34	M	Lower extremities	Proctectomy and colostomy for perianal CD	ND	Tofacitinib	ND	Improved
6	Gregory et al. (7) (2019)	ND	F	Legs and around the stoma	UC	Abdominal colectomy with end ileostomy and Hartman pouch	Tofacitinib	Infliximab	Improved
7	Choi et al. (8) (2020)	64	M	Face, scalp, trunk, arms, legs and groin	Cocaine use, atrial fibrillation	ND	Tofacitinib	Prednisone (taper), rivaroxaban (for atrial fibrillation)	Partial resolution
8	Sedano and Jairath (9) (2021)	80	F	Right hip	UC, and AA	ND	Tofacitinib	ND	Improved
9	Orfaly et al. (10) (2021)	41	F	Legs	Mixed connective tissue disorder, unspecified IBD	ND	Tofacitinib	IVIG, systemic corti costeroids (for mixed connective tissue disorder)	Partial resolution
10	Orfaly et al. (10) (2021)	58	F	Legs	RA	ND	Tofacitinib	HCQ and systemic corticosteroids (for RA)	Complete resolution
11	Orfaly et al. (10) (2021)	55	F	Legs	Psoriasis, psoriatic arthritis	ND	Tofacitinib	IVIG	Complete resolution
12	Orfaly et al. (10) (2021)	70	M	Legs	UC	ND	Tofacitinib	Prednisone (taper), dapsone	Partial resolution
13	Salmón Olav arría et al. (11) (2021)	69	F	Left knee and ankle	UC	ND	Tofacitinib	ND	Complete resolution
14	Scheinberg et al. (12) (2021)	71	F	Scalp	IgA multiple myeloma	Herpes zoster	Baricitinib	ND	Improved
15	Scheinberg et al. (12) (2021)	59	F	Leg	RA	ND	Baricitinib	ND	Healed
16	Kooybaran et al. (13) (2022)	50	F	Both legs	RA	ND	Upadacitinib	ND	Improved
17	Castro (14) (2023)	73	M	Left axilla	Metabolic syndrome and pasthistory of inactive familial Mediterranean fever	ND	Baricitinib	ND	Remission
18	Castro (14) (2023)	89	F	Legs and foot	ND	ND	Tofacitinib	ND	Healed and relapse-free
19	Van Eycken et al. (15) (2023)	65	F	Abdom	HLAB27 negative spondylarthritis (SpA)	Abdominal surgery scar	Upadacitinib	ND	Improved
20	Dos Santos et al. (16) (2023)	45	F	Legs	RA, SARS CoV-2 infection	ND	Upadacitinib	ND	Complete resolution
21	Wang et al. (17) (2024)	44	F	Left inguinal area	ND	ND	Baricitinib	ND	Healed
22	Sathyanarayana et al. (18) (2024)	70	F	Lower-limb	ND	ND	Tofacitinib	ND	Improved
23	Sathyanarayana et al. (18) (2024)	43	M	Bilateral lower-limb	ND	ND	Tofacitinib	ND	Improved
24	Sathyanarayana et al. (18) (2024)	52	F	Bilateral lower-limb	RA	ND	Tofacitinib	MTX (20 mg/week)	Improved
25	Mendolaro et al. (19) (2024)	59	F	Upper and lower limbs	CD	Colectomy and end ileostomy	Upadacitinib	ND	Improved
26	Köken Avşar et al. (20) (2024)	41	F	Right anterior leg	Enteropathic arthritis	ND	Tofacitinib	ND	Remission
27	Park et al. (21) (2024)	62	F	Lower limb	UC	ND	Upadacitinib	ND	Improved
28	Grisé et al. (22) (2024)	82	F	Lower extremities	Squamous cell carcinoma	Wide local excision	Baricitinib	ND	Improved
29	He and Tian (23) (2025)	36	F	Lower limbs	UC	ND	Upadacitinib	Schizophrenia	Healed
30	Estrella and Verallo-Rowell (24) (2025)	54	F	Right lateral ankle	UC	ND	Abrocitinib	ND	Improved
31	Gaig et al. (this case)	20	M	Face	ND	Local excision	Upadacitinib	Prednisolone	Improved

M, male; F, female; Janus kinase, JAK; ND, not described; CD, Crohn’s disease; RA, Rheumatoid arthritis; UC, ulcerative colitis; AA, alopecia areata; IVIG, Intravenous immunoglobulins; HCQ, Hydroxychloroquine; RXT, Ruxolitinib; MTX, Methotrexate; DVT, deep vein thrombosis.

## 2 Case report

**Figure 1:**
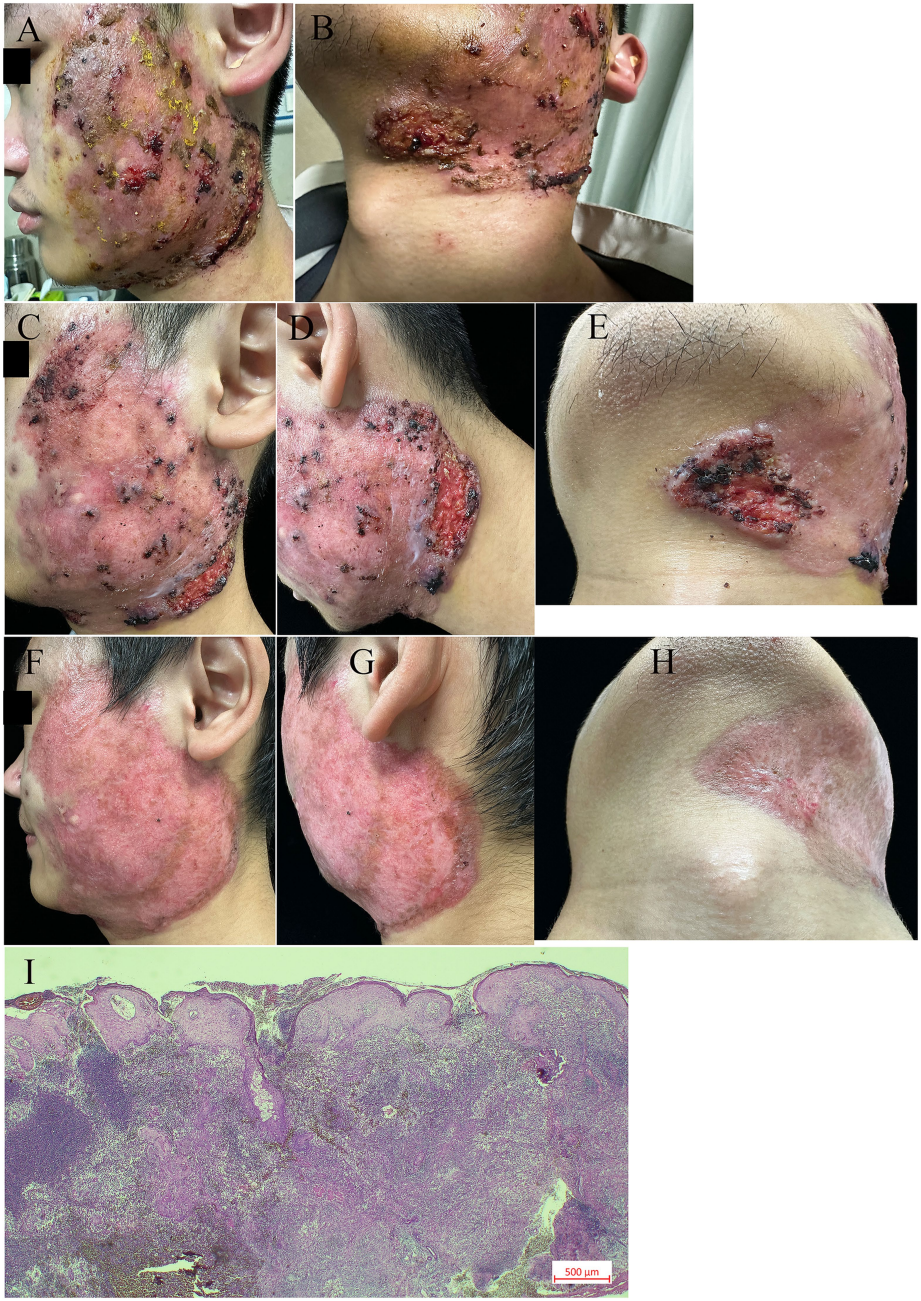
Clinical and histological features. **(A–B)** A well-demarcated dark red plaque, with local cysts present, was observed on the left side of the face. Multiple ulcers were noted on the lower edge of the red plaque, particularly near the left mandibular angle. These ulcers were surrounded by erythema, showing undermined borders, destroyed margins, and tenderness upon palpation. **(C–E)** Clinical improvement was documented at three days after the initiation of prednisolone treatment. **(F–H)** Clinical images of the left face after 24 days of pyoderma gangrenosum treatment with prednisolone and upadacitinib. **(I)** Biopsy showing diffuse mixed inflammatory cell infiltration (original magnification 10×).

We report the case of a 20-year-old man who presented with a 4-month history of painful facial skin ulcers on 18 March 2025 (Figures 1A,B). The lesion appeared after excision of facial cysts. The patient had a history of eczema and acne, with no family history of similar conditions and psychosocial issues. On 10 March 2025, a biopsy suggested an infectious granuloma; however, negative acid-fast and periodic acid–Schiff stains were negative (Figure 1I). During previous evaluations of the ulcers, next-generation sequencing suggested the presence of *Streptococcus pneumoniae* and *Klebsiella pneumoniae*; however, antimicrobial therapy was ineffective. A second biopsy was conducted on 27 March 2025. Histopathological analysis of a skin-biopsy specimen obtained from the lesion border revealed a diffuse mixed inflammatory cell infiltrate. Cultures and polymerase chain reaction tests for deep mycosis and mycobacterial infections were negative. No acid-fast bacilli, fungi, or bacteria were identified using acid-fast or periodic acid—Schiff stains. Test results for herpes simplex virus 1, herpes simplex virus 2, interferon-gamma release assays, syphilis, human immunodeficiency virus, and hepatitis B DNA all showed no abnormalities. Additionally, tests for rheumatologic conditions and immunodeficiency were negative. At the current presentation, physical examination revealed a skin ulcer with a violaceous border on the right lower portion of the face (Figures 1C–E). We made the diagnosis of facial pyoderma gangrenosum (FPG). The patient was treated with oral prednisolone (0.5 mg/kg, six tablets) monotherapy once daily for 2 weeks. However, as the ulcer enlarged, treatment was escalated to combination therapy with Upadacitinib (15 mg once daily) for 8 weeks. Treatment with Upadacitinib hydrate and a tapering course of oral prednisolone was initiated. The patient took six tablets of prednisone orally daily for 2 weeks, took four tablets for 2 weeks, and reduced the dosage by one tablet per week until the medication was discontinued. By 3 April 2025, the majority of the ulcers on the left mandible and the left neck had healed. The skin lesion started to subside 1 month after the start of treatment. Our case responded well to Upadacitinib without adverse events. After 4 months, the patient reported complete resolution of the lesions (Figures 1F–H). At present, the patient is still under our follow-up schedule.

## 3 Discussion

PG is a debilitating skin disease marked by idiopathic neutrophil infiltration that causes the destruction of tissue and ulceration (24). Epidemiological studies indicate that the average age of PG onset is in the mid-40s, with an incidence of a few cases per million person-years. PG involves dysregulation of both innate and adaptive immunity (1), leading to a neutrophil-rich autoinflammatory process with the elevation of multiple cytokines (15, 26). Some of these cytokines act through the JAK/STAT pathway (3). The importance of the JAK/STAT pathway in PG has also been demonstrated through immunohistochemistry in skin biopsy specimens (27). The predisposition of PG is not well understood. Drug induction and the postoperative period are two potential triggers. In our case, the cause of FPG is due to the excision of left-sided facial cysts.

FPG is a rare subtype of PG (25). PG needs to be differentiated from infections, such as mycobacterial cellulitis, syphilitic granulomatous ulcers, and scrofuloderma; lupus vulgaris; malignancies; and vasculitis (2). It is often associated with various other immune-mediated diseases, most commonly inflammatory bowel disease and rheumatoid arthritis (28). It may be associated with systemic inflammatory conditions, including inflammatory bowel disease (IBD), rheumatoid arthritis, or vasculitis, as well as leukemia or hepatitis. It may also be present in the setting of autoinflammatory syndromes, such as pyogenic arthritis, PG, and acne; PG, acne, and suppurative hidradenitis; and pyogenic arthritis, PG, acne, and suppurative hidradenitis, and in a small proportion of synovitis, acne, pustulosis, hyperostosis, and osteitis cases (28).

Diagnosing PG is challenging since there are no pathognomonic laboratory parameters or histopathological features (13). In 2018, a new Delphi consensus was published on the diagnostic criteria for PG, stating that diagnosis could be made by using one major and several minor criteria. The major criterion was neutrophilic infiltration at the ulcer edge on biopsy. The eight minor criteria are 1) the exclusion of infection; 2) a positive pathergy test; 3) a history of IBD or inflammatory arthritis; 4) the evolution of pustules, papules, or vesicles into ulcers within four days; 5) erythema, undermined borders, and tenderness around ulcers; 6) multiple ulcers with at least one location on the extensor surface of the lower leg; 7) cribriform or “wrinkled paper” scars at the site of healed ulcers; and 8) a reduction in ulcer size within one month after treatment with immunosuppressive drugs (29).

Treatment of PG typically starts with fast-acting immunosuppressive drugs (corticosteroids and/or cyclosporine) followed by the addition of more slow-acting immunosuppressive drugs with superior adverse event profiles, including biologics, intravenous immunoglobulin (30), and JAK inhibitors (3, 28, 31). Our case and analysis of the previously published cases demonstrate JAKi as an effective treatment option for PG (3) (Table 1). Tofacitinib (a non-selective JAK inhibitor), ruxolitinib (JAK-1/2 inhibitor), and Upadacitinib (JAK-1 inhibitor) have been reported to be successful in treating PG in a handful of reported cases (10). Patients responded in a relatively brief period of time with few reported adverse events (22).

Patients documented to be treated with JAKis (Table 1) had a mean age of 55.2 years (range: 20–89 years) and consisted of 25.8% males and 74.2% females (Table 1). PG presented as ulcerations on the lower extremities in approximately 74% (23/31) of cases. Treatments were categorized as JAKi treatment combined with concomitant medications (11/31, 35.5%) or JAKi monotherapy without concomitant medications (20/31, 64.5%). Among JAKis, the most commonly used was tofacitinib (8/20, 40.0%), followed by baricitinib (5/20, 25%) and upadacitinib (5/20, 25%). Among JAKis used with concomitant medication, tofacitinib (8/11, 72.7%) was the most common, followed by upadacitinib (2/11, 18.2%) and ruxolitinib (1/11, 9.1%). The most common concomitant medication used was systemic corticosteroids (6/11, 54.5%).

## 4 Conclusion

While the management of PG is challenging because of the lack of standardized evidence-based treatments, notable advancements are being made in its identification and management. In our case, PG initially stabilized and subsequently decreased in severity under treatment with a JAKi and a tapering course of oral prednisolone.

## Data Availability

The original contributions presented in the study are included in the article/supplementary material, further inquiries can be directed to the corresponding authors.
